# Cholinergic modulation of disorder-relevant human defensive behaviour in generalised anxiety disorder

**DOI:** 10.1038/s41398-020-01141-5

**Published:** 2021-01-05

**Authors:** Adam Perkins, Fiona Patrick, Toby Wise, Nicholas Meyer, Ndaba Mazibuko, Alice E. Oates, Anne H. M. van der Bijl, Philippe Danjou, Susan M. O’Connor, Elizabeth Doolin, Caroline Wooldridge, Deborah Rathjen, Christine Macare, Steven C. R. Williams, Allan H. Young

**Affiliations:** 1 Department of Psychological Medicine, Institute of Psychiatry, Psychology & Neuroscience, King’s College London, London, UK; 2 National Institute for Health Research Biomedical Research Centre, South London and Maudsley NHS Foundation Trust, London, UK; 3 Department of Neuroimaging, Institute of Psychiatry, Psychology & Neuroscience, King’s College London, London, UK; 4 Wellcome Trust Centre for Neuroimaging, University College London, London, UK; 5 Max Planck UCL Centre for Computational Psychiatry and Ageing Research, London, UK; 6 Department of Humanities and Social Sciences, California Institute of Technology, California, USA; 7 Department of Psychosis Studies, Institute of Psychiatry, Psychology & Neuroscience, King’s College London, London, UK; 8 South London and Maudsley NHS Foundation Trust, London, UK; 9 Faculty of Social and Behavioural Sciences, University of Leiden, Leiden, Netherlands; 10 Biotrial, Paris, France; 11 Bionomics Ltd, Adelaide, Australia; 12 BiOasis Technologies Inc., Guilford, CT USA

**Keywords:** Psychiatric disorders, Clinical pharmacology

## Abstract

Drugs that are clinically effective against anxiety disorders modulate the innate defensive behaviour of rodents, suggesting these illnesses reflect altered functioning in brain systems that process threat. This hypothesis is supported in humans by the discovery that the intensity of threat-avoidance behaviour is altered by the benzodiazepine anxiolytic lorazepam. However, these studies used healthy human participants, raising questions as to their validity in anxiety disorder patients, as well as their generalisability beyond GABAergic benzodiazepine drugs. BNC210 is a novel negative allosteric modulator of the alpha 7 nicotinic acetylcholine receptor and we recently used functional Magnetic Resonance Imaging to show it reduced amygdala responses to fearful faces in generalised anxiety disorder patients. Here we report the effect of BNC210 on the intensity of threat-avoidance behaviour in 21 female GAD patients from the same cohort. We used the Joystick Operated Runway Task as our behavioural measure, which is a computerised human translation of the Mouse Defense Test Battery, and the Spielberger state anxiety inventory as our measure of state affect. Using a repeated-measures, within-subjects design we assessed the effect of BNC210 at two dose levels versus placebo (300 mg and 2000 mg) upon two types of threat-avoidance behaviour (Flight Intensity and Risk Assessment Intensity). We also tested the effects of 1.5 mg of the benzodiazepine lorazepam as an active control. BNC210 significantly reduced Flight Intensity relative to placebo and the low dose of BNC210 also significantly reduced self-reported state anxiety. Risk Assessment Intensity was not significantly affected. Results show both human defensive behaviour and state anxiety are influenced by cholinergic neurotransmission and there provide converging evidence that this system has potential as a novel target for anxiolytic pharmacotherapy.

## Introduction

Generalised Anxiety Disorder (GAD) is characterised by chronically elevated levels of anxiety and affects millions of people worldwide^1^. Benzodiazepines are effective for acute anxiety treatment but have debilitating side effects, such as addiction and sedation^2^. Selective serotonin reuptake inhibitor antidepressants (SSRIs) are also used for anxiety treatment but suffer from slow onset of action and side effects including weight gain, sexual dysfunction, and sleepiness^3^. This situation has prompted a search for new molecules that can match or exceed the therapeutic effects of existing anxiolytics yet possess novel mechanisms of action that provide rapid relief without side effects.

One candidate molecule is the negative allosteric modulator (NAM) of the alpha 7 nicotinic acetylcholine receptor (α7 nAChR). This molecule is known as BNC210 and pre-clinical work has shown it is safe, well-tolerated and non-sedating^4^. We recently found evidence from functional Magnetic Resonance Imaging (fMRI) that BNC210 engages threat-processing brain systems, as it significantly reduced amygdala reactivity to fearful faces compared to placebo in a cohort of 24 GAD patients, as well as reducing connectivity between the amygdala and the anterior cingulate cortex^5^.

**Fig. 1:**
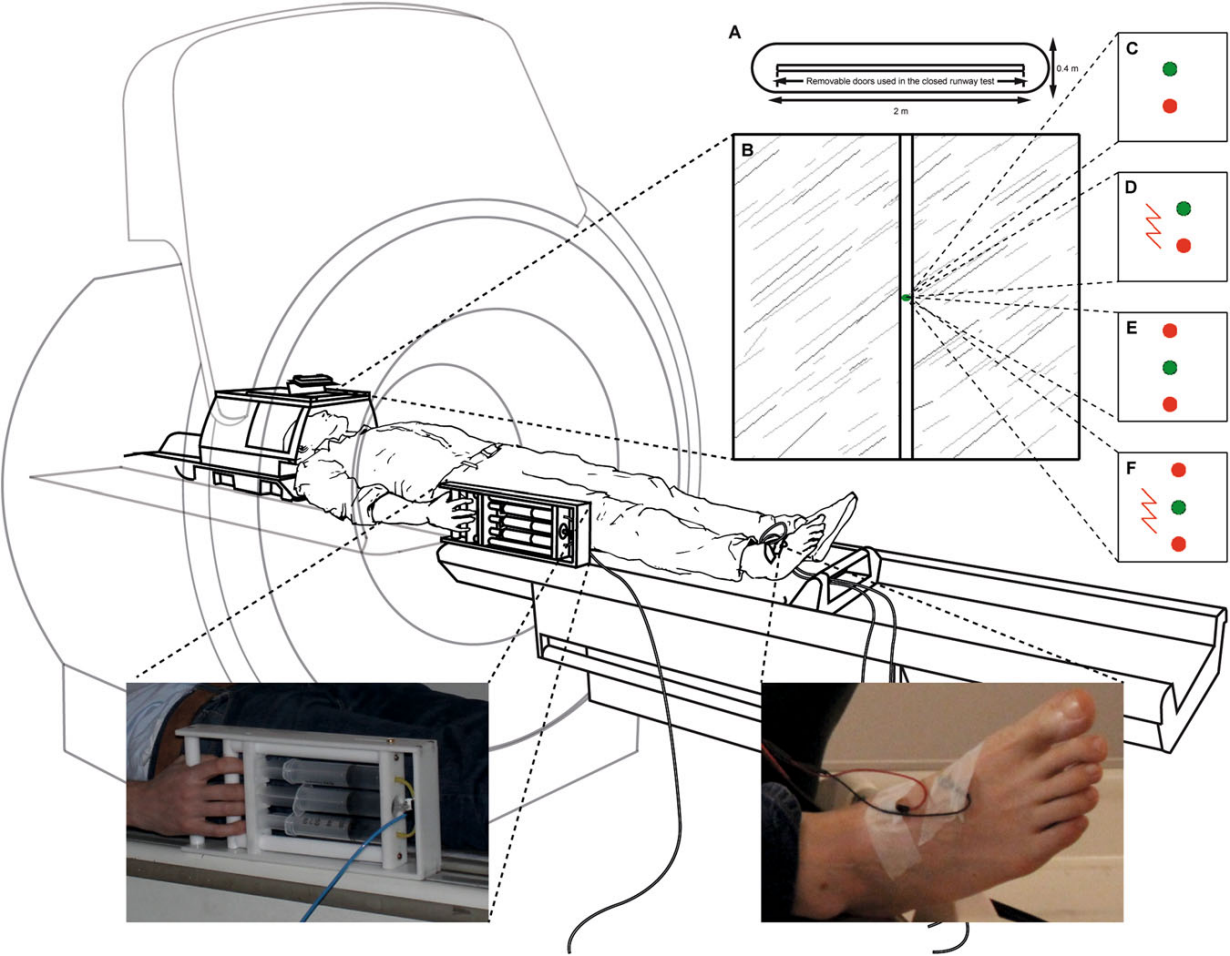
The ethoexperimental approach to measuring the intensity of threat-avoidance behaviour. **a** The Mouse Defense Test Battery. **b** The Joystick Operated Runway Task. A force-sensing interface controls the speed of a green dot cursor pursued along a runway by a red dot cursor capable of inflicting electric shock. The task comprised 12 trials each of pursuit (**c**), pursuit plus threat of electric shock (**d**), goal conflict (**e**), goal conflict plus threat of electric shock (**f**). Illustration by Nick Boon.

Here we sought behavioural evidence by measuring the effect of BNC210 on the intensity of threat-avoidance behaviour in 21 female GAD patients from the same cohort as in our fMRI study. We measured participants’ defensive behaviour using an ethoexperimental paradigm known as the Joystick Operated Runway Task (JORT)^6,7^. The JORT is a computerised human translation of the Mouse Defense Test Battery (MDTB; Fig. 1a)^8^, which is an ethoexperimental task that has been used to show that the innate defensive behaviour of rodents is sensitive to drugs with clinical effectiveness against anxiety disorders^9–11^. These rodent experiments show drugs such as SSRIs, which are clinically effective against panic disorder (PD), alter behaviour elicited by clear threat, such as running or jumping away from threat (usually labelled as flight behaviour). In contrast, drugs that are clinically effective against GAD, such as benzodiazepines, alter goal-conflict-related behaviour elicited by potential threat, such as environmental scanning and forward-backwards oscillation (usually labelled as risk assessment behaviour)^12^.

In lieu of a physical runway, the JORT combines a force-sensing joystick with an on-screen runway to measure the intensity of simple avoidance behaviour (labelled Flight Intensity; Fig. 1c, d) and two-way active avoidance behaviour (labelled Risk Assessment Intensity; Fig. 1e, f). JORT studies in healthy humans indicate human defensive behaviour is sensitive to anti-anxiety medication but have not shown the pharmacological distinction between flight and risk assessment seen in rodents. For example, in a repeated-measures, placebo-controlled study of 30 healthy human adult males, acute doses of 10 mg of the SSRI citalopram did not significantly affect Flight Intensity. However, 1 mg lorazepam significantly reduced Risk Assessment Intensity^6^. In 40 healthy adults (20 males, 20 females), contrary to predictions, there was no significant effect of 1 mg or 2 mg lorazepam on Risk Assessment Intensity but a significant dose-dependent effect of lorazepam on Flight Intensity in interaction with personality. Lorazepam reduced Flight Intensity in high scorers on the Tissue Damage Subscale of the Fear Survey Schedule but increased it in low scorers^7^. More recently, in a healthy German cohort studied after data collection ceased for the present experiment, 0.5 mg and 1 mg lorazepam did not affect Flight Intensity, whereas 0.5 mg lorazepam significantly reduced Risk Assessment Intensity relative to placebo but 1 mg lorazepam failed to do so^13^. These findings suggest the relationship between human defensive behaviour and anxiety is complex and requires further investigation. As an exploratory test of BNC210 effects on threat-avoidance behaviour, we conducted a placebo-controlled, repeated measures experiment to test the effects of two doses of BNC210 (300 mg/2000 mg) and 1.5 mg lorazepam (as an active control) on the JORT variables Flight Intensity and Risk Assessment Intensity.

## Methods

**Fig. 2:**
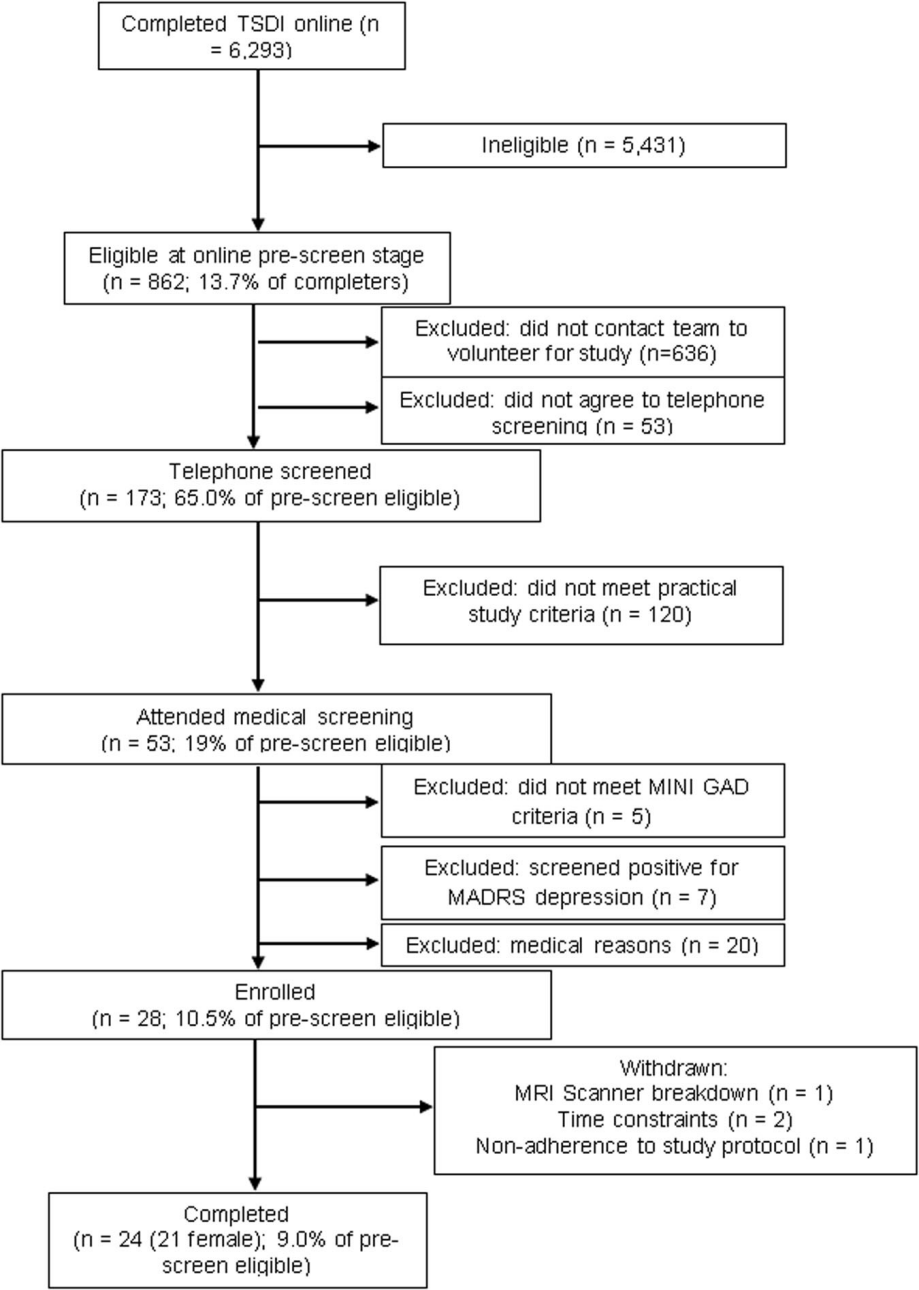
Flow chart showing the exclusion path and numbers. Abbreviations: TSDI, Trait Self-Description Inventory; MINI, Mini International Neuropsychiatric Interview; GAD, generalised anxiety disorder; MADRS, Montgomery-Asberg Depression Rating Scale; MRI, magnetic resonance imaging.

Participants were recruited according to the flow chart shown in Fig. 2. Advertisements for the study contained a weblink for a 50-item online Big Five personality questionnaire (the Trait Self-Description Inventory; TSDI) that generated a detailed personality profile for each respondent^14^. A total of 6293 people completed the questionnaire, 862 of whom met the key requirement of scoring one standard deviation or more above the mean on neuroticism as well as other study requirements (e.g., local to the study site and a non-smoker). These individuals automatically received a message accompanying their personality profile that contained information about the study and invited them to contact the research team if interested in participating. Of those 862 eligible individuals, 226 contacted the team and 173 were telephone screened, with 53 attending the study site for medical checks. The on-site checks excluded 25 of the 53 potential participants for reasons that could not be assessed during telephone screening, such as abnormal blood test results. Five candidates were excluded as they did not meet the DSM-IV criteria for GAD, which was an inclusion requirement of the study and was measured by the Mini International Neuropsychiatric Interview (MINI)^15^. To minimise co-morbidity with depression, seven participants who scored higher than 15 on the Montgomery-Asberg Depression Rating Scale (MADRS)^16^ were also excluded, as were those with any other significant psychiatric or physical illness.

This process resulted in the enrolment of twenty-eight individuals, 24 of whom completed the trial, including 21 females who are the subjects of this study (mean age 22.24 years; SD 3.52 years; age range 19–33 years). All participants provided informed consent, and the study was approved by the NRES ethics committee Chelsea, London (14/LO/2127).

This study was a repeated-measures, double-blind, randomised, placebo-controlled trial. Each participant attended four testing sessions and received either 2000 mg BNC210, 300 mg BNC210, 1.5 mg lorazepam or placebo in each visit, in a randomised order. Sessions were spaced a minimum of five days apart, to allow for pharmacological wash-out. BNC210 was orally administered as liquid suspension, with lorazepam orally administered in encapsulated tablet form. The placebo was presented either as a liquid or a capsule, depending upon which drug it was replacing. Prior to each dosing session participants had a medical health check and a standardised high fat breakfast. Tmax of BNC210 and lorazepam are approximately five hours and two hours, respectively. To maintain the blind, the dosing schedule therefore contained two dose administrations per session, with the liquid dose administered five hours before testing and the capsule dose two hours before testing. One of these doses was always placebo except in the placebo condition when both were placebo. This arrangement allowed each drug to reach Tmax around the time when the testing session began.

At enrolment, participants completed the Hamilton Anxiety Rating Scale (HAM-A)^17^. Self-report state anxiety was assessed by the state subscale of the Spielberger State-Trait Anxiety Inventory (STAI)^18^ at three timepoints; prior to first drug administration, immediately prior to JORT testing, and immediately prior to discharge at the end of the testing session (approximately seven hours after dosing). This questionnaire is a widely used self-report measure of subjective anxiety in clinical practice and has been found to be a reliable tool^19^. It comprises two scales of 20 items asking participants to report their feelings of anxiousness: the STAI State scale evaluated feelings at the time of completing the questionnaire and the STAI Trait scale evaluated how the subject generally felt. The former was administered at the three listed time points, the latter only during screening. Responses were marked on a 4-point scale from “not at all” to “very much so.” We also administered the Fear Survey Schedule (FSS)^20^ and as in previous experiments individual differences in fear-proneness modulated drug effects on defensive behaviour^7^.

JORT testing was accomplished in the supine position during fMRI scanning (Fig. 1) with a view to studying drug effects on brain activity during threat-avoidance behaviour. It should be noted however, that inability of participants to keep their heads sufficiently still in the MRI scanner while operating the joystick prevented collection of useable fMRI data.

The JORT is a computerised human translation of the MDTB^8^ (Fig. 1a) where a green dot cursor represents the participant in an on-screen runway and threat stimuli are represented by red dots. The participant used a force-sensing joystick (PH-JS14-MRI; Psyal, London, UK) to accelerate the green dot along the runway: the more force applied to the joystick, the faster the green dot cursor moved (Fig. 1b). In 50% of trials, a lightning flash icon was displayed, signifying that if the green dot was caught by a red dot, the participant would receive an electric shock. The fMRI version of the JORT delivered electric shocks to the right foot using a custom-built fMRI compatible electrical stimulator. Each participant chose a shock level they found annoying but not painful, from a choice of eight levels (maximum 80 volts at 20 mA).

The JORT measures the intensity of two types of threat-avoidance behaviour, Flight Intensity which is theoretically associated with fear and Risk Assessment Intensity which is theoretically associated with anxiety. Flight Intensity is calculated as average velocity in the one-way active avoidance trials that contained no threat of electric shock (Fig. 1c) subtracted from the average velocity in the one-way active avoidance trials with a threat of electric shock (Fig. 1d). Risk Assessment Intensity is calculated as the standard deviation of the average velocity in the two-way active avoidance trials that contained no threat of electric shock (Fig. 1e) subtracted from the standard deviation of the average velocity in the two-way active avoidance trials with threat of electric shock (Fig. 1f). The two-way active avoidance trials differ from the simple avoidance trials in that they contain a second red dot ahead of the green dot cursor, placing the participant in a goal conflict situation in which they must move fast enough to avoid the pursuing red dot but not so fast that they collide with the red dot in front. This difference in score methodology allows the JORT to control for any extraneous factors such as sedative effects of the drugs which may be present in threat and non-threat trials.

There were 12 trials for each of the four task conditions, giving a total of 48 trials. Each pursuit lasted for a maximum of seven seconds and terminated early in the event of collision between the green and the red dot. An early collision during pursuit did not reduce the total duration of the trial, hence participants could not shorten the testing session by failing to respond. To enhance unpredictability, trials were presented in pseudo-random order and intertrial intervals were varied pseudo-randomly (between 15 and 30 s). The testing procedure was done in the same order for all participants.

### Statistical analysis

In order to test for dose-response effects of BNC210 upon Flight Intensity and Risk Assessment Intensity, we conducted repeated measures ANOVAs with a three-level within-subjects factor that comprised zero dose BNC210 (placebo), low dose BNC210 (300 mg) and high dose BNC210 (2000 mg). We used previous JORT data to estimate a significant effect could be detected with approximately 20 participants. The final cohort totalled 24 participants but only female data (21 participants) were used to clarify the analysis, as there are significant sex differences in susceptibility to anxiety disorders^21,22^. Simple contrasts were used to test for the specific direction of drug effects against placebo. To benchmark BNC210 against the industry standard for anxiolytic drugs, the ANOVAs were then repeated with lorazepam added as an active control with simple contrasts again used to test for the specific direction of drug effects against placebo. Repeated measures ANOVAs with a three-level within-subjects time factor were also used to test drug effects on STAI state anxiety scores. The three time points were pre-dose, pre-JORT and pre-discharge.

## Results

### Questionnaire results

**Fig. 3:**
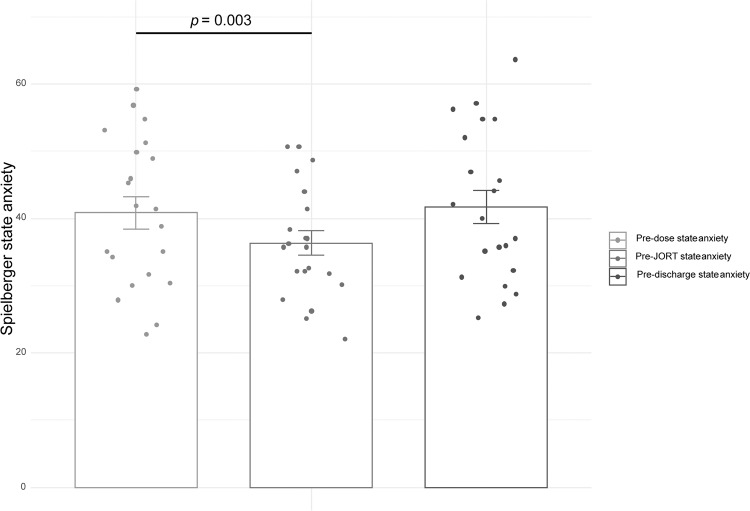
Drug effects on Spielberger State Anxiety. State anxiety was significantly decreased by the low dose of BNC210 (*n* = 21, error bars = 1 SEM).

Table 1 shows the means, SDs and intercorrelations for the HAM-A, STAI, neuroticism and FSS scores for the study cohort. The average HAM-A score placed participants in the mild-moderate range of clinical anxiety. HAM-A scores correlated significantly positively with MADRS scores, as well as FSS social fear and STAI trait anxiety but not FSS tissue damage fear. STAI state anxiety scores at the three time points (pre-dose, pre-JORT and pre-discharge) correlated significantly positively with trait anxiety scores (9 out of 12 correlations) and with each other (64 out of 66 correlations). There were no significant drug effects on STAI state anxiety scores across the three specified time points for three out of four conditions (placebo, lorazepam, high dose of BNC210): placebo F (2, 19) = 2.64, *P* = 0.084, ƞp2 = 0.122; lorazepam F (2, 19) = 1.53, *P* = 0.229, ƞp2 = 0.071; high BNC210 F (2, 19) = 2.671, *P* = 0.082, ƞp2 = 0.118. The low dose of BNC210 was the only condition to show a significant main effect of drug on STAI state anxiety: F (2, 19) = 7.53, *P* = 0.002, ƞp2 = 0.273. Simple contrasts showed that the low dose of BNC210 caused a significant reduction in state anxiety between the pre-dose administration of the STAI and the STAI administered before the JORT testing session: F (1, 20) = 11.53, *P* = 0.003, ƞp2 = 0.366. This effect became non-significant by the time the participant was discharged from the test site. F (1, 20) = 0.24, *P* = 0.630, ƞp2 = 0.012. (Fig. 3).

**Table 1:** Means, SDs and intercorrelations for questionnaire measures.

Variable	Mean	SD	1	2	3	4	5	6	7	8	9	10	11	12	13	14	15	16	17	18
1. HAM-A	16.90	9.75	—																	
2. MADRS	9.05	3.81	0.480*	—																
3. Neuroticism	71.52	5.35	0.445*	0.244	—															
4. FSS Tissue Damage Fear	32.81	19.13	0.047	0.516*	0.113	—														
5. FSS Social Fear	52.48	21.28	0.575**	0.452*	0.397	0.590**	—													
6. Trait anxiety	54.52	7.76	0.546*	0.511*	0.367	0.200	0.658**	—												
7. State anx. pre-dose pla.	42.14	11.59	0.430	0.293	0.405	0.161	0.437*	0.412	—											
8. State anx. pre-dose lor.	41.00	11.72	0.637**	0.412	0.245	0.263	0.540*	0.632**	0.687**	—										
9. State anx. pre-dose BNC L	40.86	11.04	0.398	0.250	0.460*	−0.039	0.350	0.479*	0.733**	0.650**	—									
10. State anx. pre-dose BNC H	41.43	11.14	0.585**	0.455*	0.465*	0.158	0.547*	0.621**	0.682**	0.751**	0.793**	-—								
11. State anx. pre-JORT pla.	38.43	10.68	0.782**	0.252	0.342	−0.097	0.489*	0.580**	0.767**	0.773**	0.661**	0.671**	—							
12. State anx. pre-JORT lor.	37.86	9.18	0.474*	0.372	0.340	0.134	0.597*	0.546*	0.694**	0.574**	0.653**	0.506*	0.702**	—						
13. State anx. pre-JORT BNC L	36.33	8.39	0.263	0.321	0.274	−0.073	0.261	0.416	0.538*	0.497*	0.837**	0.576**	0.532**	0.646**	—					
14. State anx. pre-JORT BNC H	37.86	10.48	0.496*	0.377	0.278	−0.039	0.433*	0.605**	0.646**	0.561**	0.652**	0.799**	0.701**	0.587**	0.572**	—				
15. State anx. pre-dis. pla.	38.20	11.59	0.698**	0.183	0.315	−0.114	0.402	0.484*	0.641**	0.695**	0.646**	0.523*	0.910**	0.750**	0.556**	0.569**	—			
16. State anx. pre-dis. lor.	38.29	9.64	0.472*	0.535*	0.125	0.121	0.506*	0.494*	0.468*	0.551**	0.572**	0.572**	0.596**	0.800**	0.754**	0.649**	0.621**	—		
17. State anx. pre-dis. BNC L	41.71	11.37	0.328	0.293	0.291	−0.264	0.121	0.352	0.592**	0.402	0.744**	0.592**	0.569**	0.512**	0.845**	0.686**	0.480*	0.574**	—	
18. State anx. pre-dis. BNC H	40.90	10.48	0.422	0.329	0.209	−0.062	0.379	0.667**	0.625**	0.607**	0.674**	0.673**	0.691**	0.679**	0.744**	0.767**	0.680**	0.691**	0.794**	—

Note. *N* = 21.

*p < 0.05. **p < 0.01.

The bold font highlights correlations that are significant at the level of *p*  < 0.01.

### Behavioural results

**Fig. 4:**
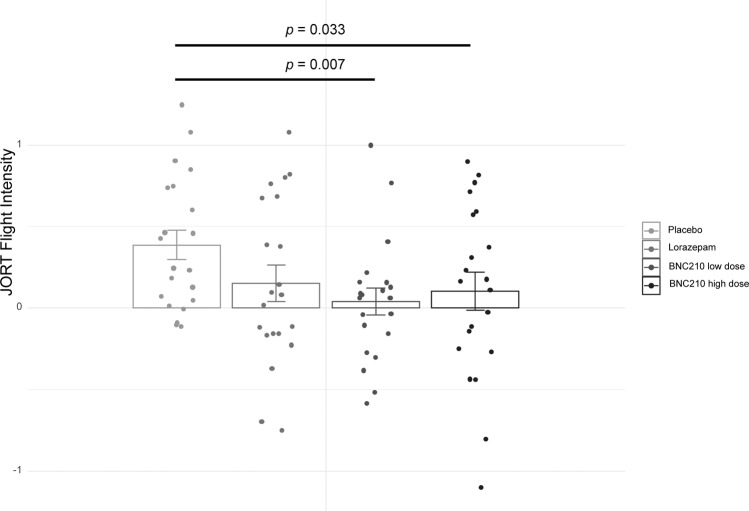
Drug effects on Flight Intensity as measured by the Joystick Operated Runway Task (JORT). Flight Intensity was significantly decreased by both low and high doses of BNC210 but not by lorazepam (*n* = 21, error bars = 1 SEM).

**Fig. 5:**
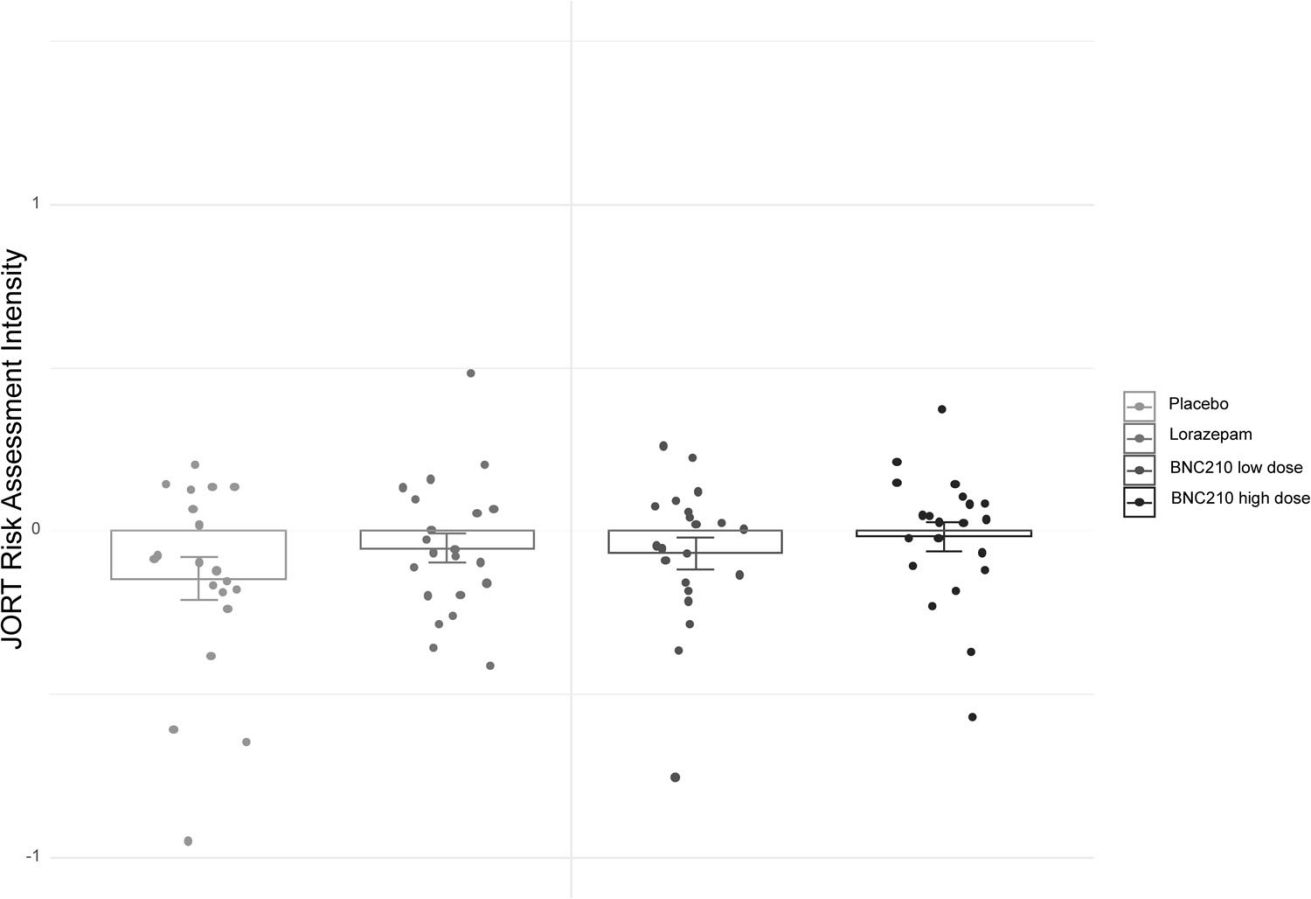
Drug effects on Risk Assessment Intensity as measured by the Joystick Operated Runway Task (JORT). Risk Assessment Intensity was not significantly altered by BNC210 or lorazepam (*n* = 21, error bars = 1 SEM).

ANOVA results show a significant main effect of drug on Flight Intensity: F (2, 19) = 4.03, *P* = 0.026, ƞp2 = 0.168. Simple contrasts showed that both low and high doses of BNC210 significantly reduced Flight Intensity relative to placebo, but that the low dose exerted a greater effect: F (1, 20) = 8.90, *P* = 0.007, ƞp2 = 0.308; F (1, 20) = 5.22, *P* = 0.033, ƞp2 = 0.207. There was no significant main effect of drug on Risk Assessment Intensity: F (2, 19) = 1.45, *P* = 0.247, ƞp2 = 0.067. The addition of lorazepam to the ANOVA reduced the main effect of drug on Flight Intensity to statistical insignificance: F (3, 18) = 1.98, *P* = 0.126, ƞp2 = 0.090. Simple contrasts showed that the effect of lorazepam on Flight Intensity failed to reach statistical significance: F (1, 20) = 2.07, *P* = 0.165, ƞp2 = 0.094 (Fig. 4). The addition of lorazepam to the ANOVA also showed no significant main effect of drug on Risk Assessment Intensity: F (3, 18) = 1.11, *P* = 0.351, ƞp2 = 0.053. Simple contrasts showed the effect of lorazepam on Risk Assessment Intensity failed to reach statistical significance: F (1, 20) = 1.43, *P* = 0.245, ƞp2 = 0.067 (Fig. 5).

## Discussion

We investigated the effect of a novel α7 nAChR NAM, BNC210, on the intensity of threat-avoidance behaviour in 21 adult female GAD sufferers using JORT. BNC210 significantly reduced Flight Intensity relative to placebo and more effectively than lorazepam. Neither drug significantly altered Risk Assessment Intensity relative to placebo. The low dose of BNC210 also significantly reduced self-reported state anxiety. The capacity of BNC210 to reduce both the intensity of threat-avoidance behaviour and self-reported state anxiety therefore provides converging evidence that it has promise as an anxiolytic drug.

In line with previous experiments that tested the effects of anxiolytics on human defensive behaviour^6,7,13^, our BNC210 results suggest it too affects brain systems that control defensive behaviour. But as in previous research, the detailed pattern of results is not as clear as rodent findings, which show a distinct behavioural/pharmacological division between panic and anxiety, with the former emotion being linked to flight behaviour and the latter to risk assessment^12^. This inter-species difference may reflect the greater cortical elaboration of humans providing a greater role for abstract thought in response to threat, due to what has been dubbed the “experiment-knowledge problem”^23^. For example, the informed consent process means that, unlike rodents, humans know that by volunteering for a JORT experiment, they will be exposed to noxious stimuli. The knowledge of the unpleasant physical events that will happen in the JORT testing room means that entering it is itself an anxiety-eliciting approach-to-threat situation for humans. Under such circumstances it is plausible that anxiolytics may affect human behaviour in JORT trial types that contain no explicit goal conflict, such as the simple avoidance trials used to measure Flight Intensity, whereas the drugs do not show such effects in rodents.

Our data also require some qualifications to be considered. Most importantly, results show both low and high doses of the candidate anxiolytic drug BNC210 significantly reduced Flight Intensity relative to placebo, but as a u-shaped relationship in which the low dose of BNC210 was most effective. This latter result echoes the findings of our recent fMRI study in this same cohort, suggesting that although it could perhaps be a cohort effect, it is not a methodological artefact, as the fMRI data showed the low dose of BNC210 was the most potent in reducing amygdala reactivity to fearful faces compared to placebo and also reduced connectivity between the amygdala and the anterior cingulate cortex^5^. In that paper we suggested that the effects of BNC210 may be explained by suppressive action on glutamatergic interneurons in the basolateral amygdala. Since we here obtained a similar pattern of results with the behavioural data, it seems likely that a similar explanation is also plausible. For example, if it is true that in GAD sufferers, the excitatory/inhibitory balance has been lost and the system contains too much glutamate and not enough GABA, then it may be the case that the 300 mg dose of BNC210 is sufficient to restore homoeostasis by decreasing glutamate release in the amygdala and hippocampus but the 2000 mg dose of BNC210 destabilises the system, returning it to the excessive glutamate situation.

A u-shaped dose response pattern is termed hormesis^24,25^ and is thought to occur when the balance of a homoeostatic system is optimised by a low dose of a drug but destabilised by either a high dose or no dose/placebo^26^. The amygdala is one of the brain regions highly regulated by cholinergic input and it has been shown in mice that activation of presynaptic nAChRs can modulate both glutamatergic (excitatory) and GABAergic (inhibitory) synaptic transmission. This modulation is sensitive to α-bungarotoxin, an α7 nAChR-specific antagonist thus suggesting a role for α7 receptors^26^. Other experiments in rats have shown that α7 nAChRs are the predominant nicotinic receptor subtype expressed on the somato-dendritic regions of a subset of amygdala neurons in the basolateral amygdala and lateral nuclei^27^. Neuroscientific work suggests these neurons form part of a group of brain regions that have been dubbed the “cognitive fear” circuitry, which controls appraisal of threat^28^.

Together these fMRI and behavioural data gathered in this GAD cohort suggest that α7 nAChRs are important for regulating excitability in regions of the amygdala that are related to perceptions of threat intensity. In the case of BNC210 in our anxious subjects, it may be that the 300 mg dose was optimal for balancing glutamatergic (excitatory) and GABAergic (inhibitory) synaptic transmission in response to threat, whereas the placebo and 2000 mg conditions were not.

Hence, viewed as a whole, the results of our two BNC210 studies, which used different methods (fMRI and JORT respectively) in the same cohort of GAD subjects, support the notion that anxiety disorders reflect altered functioning in brain systems that process threat^9–12^. They also suggest that the novel α7 nAChR negative allosteric modulator BNC210 at the 300 mg dose has potential as an anti-anxiety medication, perhaps because it restores the homoeostatic balance between glutamatergic and GABAergic systems.

This conclusion must be tempered with the finding that neither BNC210 nor lorazepam had a significant effect on Risk Assessment Intensity. This may have been a product of suboptimal dose selection, as German work published after these data were collected showed 0.5 mg lorazepam reduced Risk Assessment Intensity^13^. Given that in rodent work risk assessment behaviour is only seen at intermediate threat levels, anxiolytic drugs can increase or decrease values depending on the threat level. It is possible therefore that Risk Assessment Intensity is sensitive to lower doses of drugs than Flight Intensity and with hindsight we should have used a lower dose range for lorazepam in the present study.

Conclusions must also be tempered by the finding that self-reported state anxiety as measured by the Spielberger STAI was not affected by lorazepam or the high dose of BNC210. However, as shown in Fig. 3, the low dose of BNC210 did significantly reduce state anxiety before the JORT testing session, a finding that echoes its reducing effect on Flight Intensity. Since Spielberger state anxiety is a self-report method and the JORT is a behavioural method, the cross-methodological convergence of BNC210 effects suggests these findings are not artefactual quirks of a particular method but may be due to a genuine anxiolytic effect of BNC210, at least in the 300 mg dose.

As a caveat it should be noted that our participants were all female due to difficulties in recruiting sufficient male GAD sufferers to obtain a balanced sample. With regard to the female findings, sex differences in the neurobiology of fear have been identified, which have substantial implications for anxiety disorders^29^. Women are twice as likely to have an anxiety disorder^21,22^. Emerging evidence suggests that men and women differ in how they form conditional fear memories and extinguish fear memories^30^, hence the discovery that BNC210 reduced threat-avoidance intensity in our female participants is reassuring evidence as to the clinical utility of BNC210. Previous research into lorazepam effects on the threat-avoidance behaviour of healthy participants contained equal numbers of both sexes and did not show this sex specific effect^7^ suggesting that had we managed to recruit equal numbers of males and females the BNC210 effect may have been present in both. This situation makes a larger scale replication of the present experiment with equal numbers of males and females a matter of considerable scientific importance.

As mentioned above, previous published work by our team, using this same cohort, showed that the low dose of BNC210 reduces amygdala responses to fearful faces and reduces task-related anterior-cingulate functional connectivity in a sample of individuals with GAD^5^. Our behavioural and self-reported state anxiety data complement that work since amygdala hyperactivity to threat-related stimuli reflects anxiety^31,32^, and marketed anxiolytic drugs altering the GABAergic and serotonergic systems, reduce amygdala responses to these stimuli^33,34^. Viewed together, these findings suggest normalisation of amygdala response may underpin the clinical effects of anxiolytic medications which reduce anxiety and threat avoidance behaviour. Our research in this GAD cohort therefore provided several useful pieces of information. It established that regions of the brain that are involved in anxiety can be positively affected by BNC210, and it showed that the drug could then induce defence-related behaviour changes as demonstrated by the JORT. These results respectively provide a physiological and a functional basis to support the potential anxiolytic activity of modulators of cholinergic transmission such as BNC210.
